# Awareness of Urgent Care Services Among Primary Healthcare Center Patients in Al-Ahsa, Saudi Arabia

**DOI:** 10.7759/cureus.57099

**Published:** 2024-03-28

**Authors:** Mohammed A Almansour, Mohammed N Alhussain, Maitham N Alsarhan

**Affiliations:** 1 Family Medicine, Family Medicine Academy, Al-Hofuf, SAU

**Keywords:** primary healthcare center, awareness, family medicine, saudi arabia, utilization, urgent care clinic

## Abstract

Background

Urgent care clinics (UCCs) provide services for patients without the need to book an appointment in advance to treat acute diseases and injuries that do not need ED service and provide care for chronic conditions. This study aimed to assess the patients's level of awareness regarding UCCs in the Al-Ahsa region and provide information contributing to decision-makers' support about the urgent care services and their patterns of use within primary healthcare.

Methods

A descriptive cross-sectional study was conducted in the Al-Ahsa region from August 2023 to December 2023. A validated questionnaire was used. Cluster sampling was used to select three primary healthcare centers from four health sectors (southern cluster, middle cluster, northern cluster, and eastern cluster), and then simple random sampling was used to select participants. sample size calculated by Cochran's sample size formula, which estimated 377 participants. However, to accommodate a non-response rate of 10.0% and stronger statistical power and effect size, the final sample size was 469 participants. Data were analyzed by SPSS Statistics version 28 (IBM Corp. Released 2021. IBM SPSS Statistics for Windows, Version 28.0. Armonk, NY: IBM Corp.). Both descriptive and inferential statistics were used. A p-value ≤0.05 is considered statistically significant.

Results

Of the 469 participants in the study, more than half (54.8%) were aged between 18 and 38 years old, and more than half (54.8%) were male. More than half (67.4%) reported having no chronic diseases, whereas the most common chronic diseases reported were diabetes mellitus (11.9%) and hypertension (14.3%). Most of the participants (84.9%) weren’t aware of UCCs. Among the participants who were aware of UCCs (n=71), 53.5% of them had visited a UCC in the last three months. The most common reasons for their visits were the common cold (40.8%), headaches (5.6%), and abdominal pain (5.6%). More than one-third of participants (38.6%) believed that UCCs provide services like those of the emergency center. According to patients' sex, there was a significant (0.031) difference in the awareness level; the highest was among females at 20.1% vs. 12.6%.

Conclusion

The study revealed that the majority of the participants were unaware of UCCs. Increasing patients' awareness of UCCs is necessary through different media to improve access to healthcare services and reduce overcrowding in the ED that is caused by non-urgent problems.

## Introduction

An urgent care clinic (UCC) is defined by the American Academy of Urgent Care Medicine (AAUCM) as “the provision of immediate medical service offering outpatient care for the treatment of acute illness and injury” [1]. UCCs consider walk-in clinics, which provide healthcare services for patients without booking an appointment in advance, for a variety of diseases and injuries that are not severe enough to require ED visits [2]. In contrast, a primary healthcare center (PHC) is defined by the American Academy of Family Physicians as “the provision of integrated, accessible healthcare services by physicians and their healthcare teams who are accountable for addressing a large majority of personal healthcare needs, developing a sustained partnership with patients, and practicing in the context of family and community” [3]. UCCs can deal with several diseases, including acute and chronic cases, through a patient-centered and comprehensive approach and in connection with another medical specialty [4].

ED demand has been increasing annually by about 3% to 6% in many countries, including the United States, Canada, Australia, and the United Kingdom [5-8]. A previous study in Saudi Arabia showed increased emergency service utilization due to an increased number of patients [9,10]. The increase in the number of ED visits led to increased waiting times and costs. ED crowding is considered a worldwide issue [11-15]. A study showed that 10-60% of ED cases can be managed using lower acuity care services [16]. It takes about one hour in a UCC to provide services for walk-in patients [17]. A different study showed the main reasons for ED and UCC visits are lack of access to primary care, convenience in terms of better opening hours or being located closer to home than alternatives, and recommendations from a healthcare provider, relatives, or friends [18].

Literature shows unsatisfactory awareness regarding UCCs in Saudi Arabia. The most common cause of urgent care visits was the common cold (25.76%) [11]. In Saudi Arabia, UCCs consider a new scope of services in the new healthcare transformation strategy, which is one of the strategic goals of the Saudi Vision 2030 [4]. In Al-Ahsa, urgent care services are provided in many PHCs: Alsalhya PHC, Aljaber PHC, Eskan-Alhkabia PHC, Aloyun PHC, and Al Omran PHC.

This study aimed to assess the patients' level of awareness of UCCs in Al-Ahsa and identify the factors associated with patient awareness of UCCs.

## Materials and methods

This was a descriptive, cross-sectional, patient-based study. The study was conducted in the Al-Ahsa region, which accounts for about 20% of the Saudi Arabian area. It is considered the largest governorate in the Eastern Province of Saudi Arabia. It contains four major cities: Al-Hofuf, Al-Mubarraz, Al-Oyun, and Al-Umran. The total population of Al-Ahsa is about 1,369,338 people. Al-Ahsa is divided into four health clusters: the southern cluster, middle cluster, northern cluster, and eastern cluster, with a total of 66 PHCs, so cluster sampling was used to choose three PHCs from each sector randomly, and from each center, we selected the participants by using a simple random technique. The sample size was calculated by using a margin of error of 5%, a confidence level of 95%, and a response distribution of 50% through the following formula:

n =z2p(1 − p) /d2

where z represents the confidence level (95%), p represents the proportion of the dependent variable under study in the population (50%), and d represents the margin of error (0.05).

So, the sample size is 377. However, to accommodate a non-response rate of 10.0% and stronger statistical power and effect size, the final sample size was 469 participants.

All adult participants of Saudi nationality, older than 18 years, and not working in the health field were included in this study, while respondents were excluded if they were working in the health field, below the age of 18 years, and did not agree to participate.

Data was collected by a validated 16-item questionnaire that was used in a previous study [11]. Permission to use the questionnaire was granted by the authors, Albalahi NM. Data was collected by a validated 16-item questionnaire that was used in a previous study [11]. The questionnaire has two main parts: the first part is sociodemographic data (age, sex, marital status, educational level, employment status, family income, and history of chronic diseases); the second part is the extent of awareness and usage pattern of UCC. The participant who chose the aware option was considered aware, and those who were not were considered unaware.

Statistical analysis

Data were analyzed by SPSS Statistics version 28 (IBM Corp. Released 2021. IBM SPSS Statistics for Windows, Version 28.0. Armonk, NY: IBM Corp.). Categorical variables are presented as frequencies and percentages. The chi-square test was used for categorical variable comparison. A p-value ≤0.05 was considered statistically significant.

Ethical considerations

The study was approved by the Institutional Review Board of King Fahad Hospital - Hofuf (approval number: 99-EP-2023). Informed consent was obtained from all the participants of the study, and the participants have the right to withdraw from the study at any time. All data remained confidential. The study shows no conflict of interest.

## Results

Of the 469 participants in the study, more than half (54.8%) were aged between 18 and 38 years old, followed by 36.5% between 39 and 60, more than half (54.8%) were male, the majority (67.4%) were married, followed by single individuals (29.4%), about two-thirds had completed university education (66.1%), followed by high school graduates (22.6%). In addition, 44.6% identified as employees, followed by students (19.0%) and more than one-third (38.4%) of their family income between 6,000 and 12,000 SAR monthly, followed by more than 12,000 SAR (35.8%). A significant portion of participants (67.4%) reported having no chronic diseases, whereas the most common chronic diseases reported were hypertension (14.3%) and diabetes mellitus (11.9%). All details are shown in Table 1.

**Table 1:** Participants' demographic characteristics (n=469)

Questions	Answers	Frequency (%)
Age by years	18-38	257 (54.8%)
	39-60	171 (36.5%)
	More than 60	41 (8.7%)
Sex	Male	257 (54.8%)
	Female	171 (36.5%)
Marital status	Single	138 (29.4%)
	Married	316 (67.4%)
	Divorced	8 (1.7%)
	Widowed	7 (1.5%)
Educational level	Primary stage	4 (.9%)
	Intermediate stage	13 (2.8%)
	High school	106 (22.6%)
	University stage	310 (66.1%)
	Postgraduate studies	26 (5.5%)
	Diploma	10 (2.1%)
Employment status	Employee	209 (44.6%)
	Student	89 (19.0%)
	Retired	70 (14.9%)
	Unemployed	101 (21.5%)
Family income	<6,000 SAR	121 (25.8%)
	6,000 to 12,000 SAR	180 (38.4%)
	More than 12,000 SAR	168 (35.8%)
Chronic diseases	Diabetes mellitus	56 (11.9%)
	Hypertension	67 (14.3%)
	Hypercholesterolemia	29 (6.2%)
	Thyroid disorders	16 (3.4%)
	Asthma	24 (5.1%)
	Others	28 (6.0%)
	No	316 (67.4%)

The awareness and use of urgent care services are shown in Table 2. Most of the participants (84.9%) had not heard about UCCs; among the participants who were aware of UCCs (n=71), 46.5% reported not having visited a UCC in the last three months. Of those who did visit, 35.2% had one to three visits, 8.5% had four to six visits, and 9.9% had more than six visits. The most common reasons for their visit were the common cold (40.8%), followed by headaches (5.6%) and abdominal pain (5.6%).

**Table 2:** Usage and awareness of urgent healthcare services

Questions	Answers	Frequency (%)
Have you ever heard of the term urgent care clinics?	Yes	71 (15.1%)
	No	398 (84.9%)
How many visits to the urgent care clinics during the three A last month? (n=71)	0	33 (46.5%)
	1‑3	25 (35.2%)
	4‑6	6 (8.5%)
	More than 6	7 (9.9%)
Was the reason for your visit to the urgent care clinic one of the following reasons? (n=71)	Common cold	29 (40.8%)
	Abdominal pain	4 (5.6%)
	Chest pains	1 (1.4%)
	Lower back pain	2 (2.8%)
	Bone fractures	2 (2.8%)
	Headache	4 (5.6%)
	Something else	6 (8.5%)
	No visit	23 (32.4%)
Have you ever visited the primary healthcare centers without booking an appointment in advance?	Yes	197 (42.0%)
	No	272 (58.0%)
What was the reason for visiting the primary healthcare centers without an appointment? (n=197)	Having difficulty in booking appointments	92 (46.7%)
	A recent health problem	95 (48.2%)
	Other reasons	10 (5.1%)
Do you think that the urgent care clinics provide services similar to those of the emergency center?	Yes	181 (38.6%)
	No	51 (10.9%)
	I do not know	237 (50.5%)
In your opinion, what are the categories allowed to visit urgent care clinics?	Children	297 (63.3%)
	Males	150 (32.0%)
	Women	179 (38.2%)
	Elderly	317 (67.6%)
	Pregnant women	236 (50.3%)
	Domestic workers	109 (23.2%)
	Health center employees	84 (17.9%)
	People with special needs	238 (50.7%)
	I don't know	128 (27.3%)
What do you think is the main function of the urgent care clinics?	Treatment of chronic conditions) such as blood pressure, diabetes, asthma, etc.)	252 (53.7%)
	Refill medicine	122 (26.0%)
	Treatment of acute symptoms that do not require going to the emergency center	253 (53.9%)
	Detecting health conditions that entail emergent intervention	286 (61.0%)
	Helping struggling cases to get an appointment in public clinics	238 (50.7%)
	Follow‑up	89 (19.0%)
	Something else	7 (1.5%)

When asked if they had ever visited PHCs without a prior appointment, 42.0% of participants responded affirmatively. The reasons for attending PHCs without an appointment were having trouble obtaining appointments (46.7%), developing a new health problem (48.2%), and other unspecified reasons (5.1%).

More than one-third of participants (38.6%) believed that UCCs provide services like those of the emergency center. The majority believed that children (63.3%), the elderly (67.6%), pregnant women (50.3%), and people with special needs (50.7%) were allowed to visit UCCs. Other categories mentioned included males (32.0%), women (38.2%), domestic workers (23.2%), and health center employees (17.9%). A significant percentage (27.3%) responded that they did not know.

The majority of participants (61.0%) believe that UCCs are primarily for detecting health conditions that require emergent intervention, treatment of acute symptoms that do not require going to the emergency center (53.9%), treatment of chronic conditions (53.7%), helping struggling cases get an appointment in public clinics (50.7%), refill medicine (26.0%), and follow-up (19.0%).

Table 3 displays the awareness and use of UCC services by gender. The findings indicate that there is a significant variation in the awareness of UCCs between males and females, with 12.6% of males and 20.1% of females reporting awareness with a p-value of 0.031. However, there were no significant differences between genders in terms of the frequency of visits to UCCs and reasons for visiting or going to PHCs without making a reservation. Concerning those who are permitted to attend UCCs, females reported that the elderly (76.7% vs. 62.9%), children (76.1% vs. 56.8%), people with special needs (59.7% vs. 46.1%), and pregnant women (57.9% vs. 46.5%), respectively. Concerning the primary goal of UCCs, a higher percentage of females (69.8%) than males (56.5%) believed that UCCs identify medical problems that need emergency care, with a p-value of 0.005.

**Table 3:** Gender differences in awareness and usage of urgent care services *: Significant

Questions	Answers	Sex				p-value
		Male		Female		
		N	%	N	%	
Have you ever heard of the term urgent care clinics?	Yes	39	12.60%	32	20.10%	0.031*
	No	271	87.40%	127	79.90%	
How many visits to the urgent care clinics during the three A last month?	0	18	46.20%	15	46.90%	0.993
	1‑3	14	35.90%	11	34.40%	
	4‑6	3	7.70%	3	9.40%	
	More than 6	4	10.30%	3	9.40%	
Was the reason for your visit to the urgent care clinic one of the following reasons?	Common cold	18	46.20%	11	34.40%	0.877
	Abdominal pain	2	5.10%	2	6.30%	
	Chest pains	1	2.60%	0	0.00%	
	Lower back pain	1	2.60%	1	3.10%	
	Bone fractures	1	2.60%	1	3.10%	
	Headache	2	5.10%	2	6.30%	
	Something else	4	10.30%	2	6.30%	
	No visit	10	25.60%	13	40.60%	
Have you ever visited the primary healthcare centers without booking an appointment in advance?	Yes	132	42.60%	65	40.90%	0.724
	No	178	57.40%	94	59.10%	
What was the reason for visiting the primary healthcare centers without an appointment?	Having difficulty booking appointments	62	47.00%	30	46.20%	0.967
	A recent health problem	63	47.70%	32	49.20%	
	Other reasons	7	5.30%	3	4.60%	
Do you think that the urgent care clinics provide services similar to those of the emergency center?	Yes	105	33.90%	76	47.80%	0.002*
	No	42	13.50%	9	5.70%	
	I do not know	163	52.60%	74	46.50%	
In your opinion, what are the categories allowed to visit urgent care clinics?	Children	176	56.80%	121	76.10%	<0.000*
	Males	99	31.90%	51	32.10%	0.975
	Women	110	35.50%	69	43.40%	0.095
	Elderly	195	62.90%	122	76.70%	0.002*
	Pregnant women	144	46.50%	92	57.90%	0.019*
	Domestic workers	65	21.00%	44	27.70%	0.104
	Health center employees	46	14.80%	38	23.90%	0.015*
	People with special needs	143	46.10%	95	59.70%	0.005*
	I do not know	99	31.90%	29	18.20%	0.002*
What do you think is the main function of the urgent care clinics?	Treatment of chronic conditions) such as blood pressure, diabetes, asthma, etc.)	166	53.50%	86	54.10%	0.912
	Refill medicine	86	27.70%	36	22.60%	0.223
	Treatment of acute symptoms that do not require going to the emergency center	168	54.20%	85	53.50%	0.88
	Detecting health conditions that entail emergent intervention	175	56.50%	111	69.80%	0.005*
	Helping struggling cases to get an appointment in public clinics	159	51.30%	79	49.70%	0.742
	Follow‑up	63	20.30%	26	16.40%	0.299
	Something else	7	2.30%	0	0.00%	0.056

The difference in awareness and utilization by participants’ age is shown in Table 4. A study reported that individuals from different age groups had varying reasons for attending PHCs without a scheduled meeting. Among the age group of 18-38, a recent health problem was mentioned, whereas in the age group of 39-60, more than 60% reported having difficulty making appointments at 54.5% and 47.8%, respectively (p=0.009). Regarding the groups allowed to attend UCCs, there were no significant differences observed among the age groups, except for pregnant women, people with special needs, health center employees, and males.

**Table 4:** Utilization and awareness of urgent care clinics by age group *: Significant

Questions	Answers	Age						p-value
		18 -38		39 - 60		More than 60		
		N	%	N	%	N	%	
Have you ever heard of the term urgent care clinics?	Yes	35	13.60%	29	17.00%	7	17.10%	0.6
	No	222	86.40%	142	83.00%	34	82.90%	
How many visits to the urgent care clinics during the three A last month?	0	18	51.40%	11	37.90%	4	57.10%	0.802
	1‑3	11	31.40%	11	37.90%	3	42.90%	
	4‑6	3	8.60%	3	10.30%	0	0.00%	
	More than 6	3	8.60%	4	13.80%	0	0.00%	
Was the reason for your visit to the urgent care clinic one of the following reasons?	Common cold	14	40.00%	12	41.40%	3	42.90%	0.259
	Abdominal pain	2	5.70%	2	6.90%	0	0.00%	
	Chest pains	1	2.90%	0	0.00%	0	0.00%	
	Lower back pain	0	0.00%	1	3.40%	1	14.30%	
	Bone fractures	1	2.90%	1	3.40%	0	0.00%	
	Headache	1	2.90%	1	3.40%	2	28.60%	
	Something else	2	5.70%	3	10.30%	1	14.30%	
	No visit	14	40.00%	9	31.00%	0	0.00%	
Have you ever visited the primary healthcare centers without booking an appointment in advance?	Yes	110	42.80%	69	40.40%	18	43.90%	0.852
	No	147	57.20%	102	59.60%	23	56.10%	
What was the reason for visiting the primary healthcare centers without an appointment?	Having difficulty booking appointments	49	44.50%	33	47.80%	10	55.60%	0.009*
	A recent health problem	60	54.50%	30	43.50%	5	27.80%	
	Other reasons	1	0.90%	6	8.70%	3	16.70%	
Do you think that the urgent care clinics provide services similar to those of the emergency center?	Yes	104	40.50%	63	36.80%	14	34.10%	0.073
	No	36	14.00%	12	7.00%	3	7.30%	
	I do not know	117	45.50%	96	56.10%	24	58.50%	
In your opinion, what are the categories allowed to visit urgent care clinics?	Children	174	67.70%	100	58.50%	23	56.10%	0.092
	Males	97	37.70%	44	25.70%	9	22.00%	0.012
	Women	106	41.20%	63	36.80%	10	24.40%	0.108
	Elderly	174	67.70%	111	64.90%	32	78.00%	0.271
	Pregnant women	150	58.40%	67	39.20%	19	46.30%	<0.001*
	Domestic workers	70	27.20%	31	18.10%	8	19.50%	0.077
	Health center employees	60	23.30%	19	11.10%	5	12.20%	0.003*
	People with special needs	150	58.40%	71	41.50%	17	41.50%	0.001*
	I do not know	66	25.70%	53	31.00%	9	22.00%	0.349
What do you think is the main function of the urgent care clinics?	Treatment of chronic conditions (such as blood pressure, diabetes, asthma, etc.)	143	55.60%	87	50.90%	22	53.70%	0.626
	Refill medicine	61	23.70%	47	27.50%	14	34.10%	0.138
	Treatment of acute symptoms that do not require going to the emergency center	145	56.40%	85	49.70%	23	56.10%	0.378
	Detecting health conditions that entail emergent intervention	159	61.90%	106	62.00%	21	51.20%	0.407
	Helping struggling cases to get an appointment in public clinics	134	52.10%	85	49.70%	19	46.30%	0.744
	Follow‑up	57	22.20%	27	15.80%	5	12.20%	0.131
	Something else	6	2.30%	1	0.60%	0	0.00%	0.244

As shown in Table 5, regarding categories allowed to visit UCCs, a significantly higher percentage of high educational level participants reported the elderly (70.5% vs. 60.2%), pregnant women (56.0% vs. 36.1%), people with special needs (55.4% vs. 39.1%), women (42.0% vs. 28.6%), males (35.4% vs. 23.3%), domestic workers (26.8% vs. 14.3%), and health center employees (21.1% vs. 9.8%), respectively.

**Table 5:** Utilization and awareness of urgent care services correlated with level of education *: Significant

Questions	Answers	Educational level				p-value
		High school or less		University and above		
		N	%	N	%	
Have you ever heard of the term urgent care clinics?	Yes	19	14.30%	52	15.50%	0.764
	No	114	85.70%	284	84.50%	
How many visits to the urgent care care clinics during the three A last month?	0	5	26.30%	28	53.80%	0.17
	1‑3	9	47.40%	16	30.80%	
	4‑6	3	15.80%	3	5.80%	
	More than 6	2	10.50%	5	9.60%	
Was the reason for your visit to the urgent care clinic one of the following reasons?	Common cold	8	42.10%	21	40.40%	0.133
	Abdominal pain	3	15.80%	1	1.90%	
	Chest pains	1	5.30%	0	0.00%	
	Lower back pain	1	5.30%	1	1.90%	
	Bone fractures	0	0.00%	2	3.80%	
	Headache	1	5.30%	3	5.80%	
	Something else	2	10.50%	4	7.70%	
	No visit	3	15.80%	20	38.50%	
Have you ever visited the primary healthcare centers without booking an appointment in advance?	Yes	52	39.10%	145	43.20%	0.422
	No	81	60.90%	191	56.80%	
What was the reason for visiting the primary healthcare centers without an appointment?	Having difficulty booking appointments	19	36.50%	73	50.30%	0.087
	A recent health problem	28	53.80%	67	46.20%	
	Other reasons	5	9.60%	5	3.40%	
Do you think that the urgent care clinics provide services similar to those of the emergency center?	Yes	42	31.60%	139	41.40%	0.146
	No	16	12.00%	35	10.40%	
	I do not know	75	56.40%	162	48.20%	
In your opinion, what are the categories allowed to visit urgent care clinics?	Children	77	57.90%	220	65.50%	0.125
	Males	31	23.30%	119	35.40%	0.011*
	Women	38	28.60%	141	42.00%	0.007*
	Elderly	80	60.20%	237	70.50%	0.030*
	Pregnant women	48	36.10%	188	56.00%	<0.001*
	Domestic workers	19	14.30%	90	26.80%	0.004*
	Health center employees	13	9.80%	71	21.10%	0.004*
	People with special needs	52	39.10%	186	55.40%	0.002*
	I do not know	45	33.80%	83	24.70%	0.045*
What do you think is the main function of the urgent care clinics?	Treatment of chronic conditions (such as blood pressure, diabetes, asthma, etc.)	72	54.10%	180	53.60%	0.912
	Refill medicine	34	25.60%	88	26.20%	0.889
	Treatment of acute symptoms that do not require going to the emergency center	65	48.90%	188	56.00%	0.166
	Detecting health conditions that entail emergent intervention	74	55.60%	212	63.10%	0.136
	Helping struggling cases to get an appointment in public clinics	66	49.60%	172	51.20%	0.76
	Follow‑up	24	18.00%	65	19.30%	0.746
	Something else	3	2.30%	4	1.20%	0.391

The only statistically significant variation between income categories was observed in who was eligible to visit PHCs; pregnant women are the only group mentioned (Table 6).

**Table 6:** Awareness and utilization of urgent care services by family monthly income *: Significant

Questions	Answers	Family monthly income						p-value
		<6,000 SR		6,000 to 12,000 SR		More than 12,000 SR		
		N	%	N	%	N	%	
Have you ever heard of the term urgent care clinics?	Yes	14	11.60%	24	13.30%	33	19.60%	0.116
	No	107	88.40%	156	86.70%	135	80.40%	
How many visits to the urgent care clinics during the three A last month?	0	7	50.00%	7	29.20%	19	57.60%	0.189
	1‑3	4	28.60%	9	37.50%	12	36.40%	
	4‑6	1	7.10%	4	16.70%	1	3.00%	
	More than 6	2	14.30%	4	16.70%	1	3.00%	
Was the reason for your visit to the urgent care clinic one of the following reasons?	Common cold	5	35.70%	13	54.20%	11	33.30%	0.7
	Abdominal pain	1	7.10%	2	8.30%	1	3.00%	
	Chest pains	0	0.00%	0	0.00%	1	3.00%	
	Lower back pain	1	7.10%	1	4.20%	0	0.00%	
	Bone fractures	0	0.00%	1	4.20%	1	3.00%	
	Headache	1	7.10%	2	8.30%	1	3.00%	
	Something else	1	7.10%	1	4.20%	4	12.10%	
	No visit	5	35.70%	4	16.70%	14	42.40%	
Have you ever visited the primary healthcare centers without booking an appointment in advance?	Yes	53	43.80%	70	38.90%	74	44.00%	0.558
	No	68	56.20%	110	61.10%	94	56.00%	
What was the reason for visiting the primary healthcare centers without an appointment?	Having difficulty booking appointments	20	37.70%	38	54.30%	34	45.90%	0.073
	A recent health problem	32	60.40%	30	42.90%	33	44.60%	
	Other reasons	1	1.90%	2	2.90%	7	9.50%	
Do you think that the urgent care clinics provide services similar to those of the emergency center?	Yes	46	38.00%	73	40.60%	62	36.90%	0.954
	No	13	10.70%	18	10.00%	20	11.90%	
	I do not know	62	51.20%	89	49.40%	86	51.20%	
In your opinion, what are the categories allowed to visit urgent care clinics?	Children	85	70.20%	106	58.90%	106	63.10%	0.134
	Males	36	29.80%	56	31.10%	58	34.50%	0.658
	Women	50	41.30%	60	33.30%	69	41.10%	0.235
	Elderly	84	69.40%	114	63.30%	119	70.80%	0.289
	Pregnant women	65	53.70%	76	42.20%	95	56.50%	0.019*
	Domestic workers	26	21.50%	39	21.70%	44	26.20%	0.528
	Health center employees	24	19.80%	25	13.90%	35	20.80%	0.196
	People with special needs	67	55.40%	80	44.40%	91	54.20%	0.096
	I do not know	30	24.80%	58	32.20%	40	23.80%	0.146
What do you think is the main function of the urgent care clinics?	Treatment of chronic conditions (such as blood pressure, diabetes, asthma, etc.)	74	61.20%	95	52.80%	83	49.40%	0.134
	Refill medicine	35	28.90%	39	21.70%	48	28.60%	0.238
	Treatment of acute symptoms that do not require going to the emergency center	63	52.10%	95	52.80%	95	56.50%	0.695
	Detecting health conditions that entail emergent intervention	71	58.70%	103	57.20%	112	66.70%	0.164
	Helping struggling cases to get an appointment in public clinics	64	52.90%	85	47.20%	89	53.00%	0.484
	Follow‑up	24	19.80%	29	16.10%	36	21.40%	0.433
	Something else	1	0.80%	5	2.80%	1	0.60%	191

## Discussion

This is a cross-sectional study conducted in Saudi Arabia from August 2023 to December 2023. We aimed to evaluate patients' awareness of urgent care services and their patterns of use within primary healthcare. Our findings provide valuable insights into the current state of awareness regarding urgent care services. Saudi Arabia's healthcare system is undergoing a transformation in line with the country's 2030 vision, which aims to improve access to healthcare, modernize facilities and equipment, implement telemedicine consultations, utilize data-driven treatment plans, and enhance traffic safety to meet the needs of every member of society [4].

The present study reported that most of the participants (84.9%) had not heard about UCCs. More than half of the participants who were aware of UCCs (n=35, 53.5%) visited a UCC in the last three months. The most common reasons for visits to UCC were the common cold (40.8%), headache (5.6%), and abdominal pain (5.6%). These findings are consistent with a previous study in Saudi Arabia that reported that about three-quarters (74.65%) of the patients were unaware of UCCs; 53.42% visited UCCs; and the common cold was the primary cause of the visits (25.76%) [11]. The literature revealed that although there is access to free medical care through PHCCs throughout Saudi Arabia, the most common complaints to the ED were non-urgent problems such as abdominal pain (29%), ear, eye, nose, and throat pain (23.9%), limb/joint pain (17.7%), headache (13.5%), back pain (10.4%), cold and flu symptoms (9.4%), and fever (9.1%) [19]. Insufficient community awareness of the role of the UUC and perceptions of its role lead to increased overcrowding in the ED. Increasing utilization of emergency care for non-urgent problems leads to overcrowding that is associated with increased waiting time, medical errors, patient mortality, and hospital length of stay, as well as reduced patient satisfaction and quality of care [20-22].

In the present study, 42.0% of participants visited PHCs without a scheduled visit. Having trouble scheduling appointments was the main cause of people attending PHCs without appointments (46.7%) and a recent health problem (48.2%). A study by Albalahi et al. found that almost half (45%) of patients visited PHCs without scheduling an appointment beforehand. The primary reason given for this was difficulty booking appointments (54.55%) [11]. Urgent care services can play a significant role in supporting PHCs by addressing unscheduled patient appointments with the expectation that patients will subsequently follow up with their PHCs. This indicates that urgent care is fulfilling a need for convenience and may be supplanting primary care for specific acute concerns [2].

In the current study, more than one-third of participants (38.6%) thought that services offered by UCCs were equivalent to those of emergency centers. The majority believed that children (63.3%), the elderly (67.6%), pregnant women (50.3%), and people with special needs (50.7%) were permitted to attend UCCs. Most participants (61.0%) believed that UCCs are primarily for identifying medical conditions that require immediate care and treating acute symptoms that don't require visiting the ED (53.9%), treating chronic diseases (53.7%), and assisting individuals with challenges in obtaining appointments at public clinics (50.7%). The AAUCM defines urgent care medicine as the delivery of immediate medical services across all age groups, addressing both acute and chronic illness and injury. After initially diagnosing chronic conditions, patients are then referred to primary care for follow-up [1].

Our findings indicate that females have more awareness of UCCs than males. However, there were no significant differences between genders in terms of the frequency of visits to UCCs and reasons for visiting. A review revealed that women are more aware than men regarding UCCs as well as more likely to use health services [11,23,24].

It is worth mentioning that the participants with different personal characteristics mentioned the elderly, children, pregnant women, and people with special needs as the main groups allowed to attend UCCs. Urgent healthcare service delivery of immediate medical services for all age groups [1].

Limitation

This study offers valuable information on patients’ understanding of urgent care. However, its generalizability is restricted due to the participants' specific geographic location, potentially leading to inaccurate representations of UCC usage in different areas.

## Conclusions

The study concluded that most of the participants were unaware of UCCs. More than half of participants who were aware of UCCs visited a UCC in the last three months. The most common reasons for a visit to UCC were the common cold, headache, and abdominal pain. Females were more aware of UCCs than males. Further research is needed at the national level to explore the population's awareness of UCC. Focus awareness programs on simple language by different media are needed to improve access to healthcare services and reduce overcrowding in the ED that is caused by non-urgent problems.
